# The Body Confident Mums challenge: a feasibility trial and qualitative evaluation of a body acceptance program delivered to mothers using Facebook

**DOI:** 10.1186/s12889-021-11126-8

**Published:** 2021-06-02

**Authors:** Krystina Wallis, Ivanka Prichard, Laura Hart, Zali Yager

**Affiliations:** 1 School of Psychology, University of Queensland, Queensland St Lucia, Australia; 2 Caring Futures Institute, College of Nursing and Health Sciences, Flinders University, Sturt Rd, Bedford Park, South Australia Australia; 3 Centre for Mental Health, Melbourne School of Population Health and Global Health, University of Melbourne, Parkville, Victoria Australia; 4 School of Psychology and Population Health, La Trobe University, Bundoora, Victoria Australia; 5 Institute for Health and Sport, Victoria University, Footscray Park, Victoria Australia

**Keywords:** Postpartum, Body dissatisfaction, Body image, Intervention, Mothers, Self-compassion

## Abstract

**Background:**

Motherhood is a time of intense physical, psychological, and identity transformation, and body dissatisfaction may emerge through the process of pregnancy, birth, and adaptation to parenting. We present a feasibility trial of the Body Confident Mums Challenge, a program developed by adapting existing, effective interventions that focus on self-compassion and appreciation of body functionality to be specific to mothers.

**Methods:**

The program was delivered using the social learning function in a closed Facebook group. Qualitative evaluation of evidence of change was conducted by gathering individual written reflections posted during the challenge (*n* = 120). Feasibility and acceptability was determined using a feedback survey (*n* = 22).

**Results:**

Participant’s reflective posts indicated that they were embracing self-compassion, and de-prioritising body image concerns during the challenge. Feedback indicated that the program was mostly feasible and acceptable for mothers, with recommendations from some participants relating to slowing the pace of content delivery and reducing the time commitment of the Challenge.

**Conclusions:**

The social media environment may therefore be a useful setting in which to implement brief intervention programs to improve body image and wellbeing.

## Background

Body dissatisfaction is pervasive with 91% of women indicating that they prefer an alternative body size or shape [1], and this dissatisfaction remains relatively stable across the lifespan [2]. Body dissatisfaction is linked to poor psychosocial outcomes including low mood and disordered eating [3, 4], reduced self-esteem [5, 6], and quality of life [7]. Additionally, individuals who experience body dissatisfaction are less likely to engage in health behaviours such as physical activity [3], smoking cessation [8], and skin cancer prevention behaviours [9]. Conversely, individuals that report a positive view of self and positive body image (i.e. body appreciation) are more likely to participate in health behaviours [10], and prioritise health behaviour change in their life [11]. These individuals tend to treat their body with self-compassion and respect, demonstrating increased appreciation for how their body functions whilst actively minimising perceived body imperfections [12–14]. Body appreciation is also associated with higher self-esteem, reduced depressive symptoms, intuitive eating behaviours and is a predictor for life satisfaction and long-term wellbeing [15–17].

### Body image in mothers

Pregnancy and the post-partum period represent a unique stage where women face major changes to the appearance and function of their body [18]. Research suggests pregnant women may feel absolved of their previous adherence to societal expectations of thinness during this period, given that during pregnancy it is normal and even expected to gain weight, thus reducing body dissatisfaction and drive for thinness [19]. However, any temporary gains in body satisfaction are likely to dissipate following birth where new mothers often feel pressure to ‘bounce-back’ to their pre-baby body weight and shape, but often carry more weight and feel more physically compromised after giving birth [18, 20]. This is particularly the case in the first year postpartum [21]. However, some research indicates that mothers of younger children (0–5 years) might have higher levels of body appreciation than mothers of older children (6–10 years) or women without children [22]. Despite this research, mothers with younger children are still particularly vulnerable to negative self-evaluation, and report feeling concerned about their new appearance, which may no longer be perceived to meet society’s expectations [23]. In the context of motherhood, these factors negatively impact body image and contribute to significant psychological distress and for some women, symptoms of post-partum depression [24, 25]. Research indicates other factors that may influence body image during the ante or post-natal period include the age of the mother, body mass index (BMI), weight prior to falling pregnant and previous disordered eating behaviours [21, 23].

### Successful approaches to body image interventions for adult women

According to the Tripartite model [26, 27], media, peers, and family (including partners) together facilitate internalisation of the thin-ideal, which consequently influences body dissatisfaction. This model has also been confirmed among postpartum women, and provides a platform to develop interventions which target body dissatisfaction. Mediating the internalisation of the thin-ideal, providing individuals with a buffer against broader outcomes associated with body dissatisfaction, including drive for thinness, offer a possible route to improving body image at the individual level [28].

Body image interventions for adult women have progressed significantly over the past decade. To date, approaches based on Cognitive Dissonance Theory [29] have been most successful in reducing internalisation of the thin-ideal [30], and, the counter-attitudinal advocacy component of this approach seems to be the strongest influence on inducing cognitive dissonance, and creating positive effects [31]. The Body Project [32, 33] remains the program with the highest level of evidence for adult women who identify as having body image concerns.

More recent interventions for adult women feature core themes found in third-wave therapies (i.e. gratitude, body appreciation, mindfulness, and self-compassion). There is now good evidence that leveraging aspects of these approaches can improve overall body appreciation and satisfaction [13, 34–40], and lead to overall improvements in mental health outcomes [41–43].

### E-health, M-health, and interventions on social media

Interventions that include self-compassion, mindfulness, and practicing gratitude with a focus on appreciating body functionality embed mechanisms that can be used together as complimentary approaches to improve body image [44]. However, based on a search of the literature, there are currently limited opportunities for delivery of these evidence-based resources on a large scale to communities of women, and especially mothers. Electronic health promotion programs offer a unique medium in which to promote health behaviour change, offering increased accessibility, lowered costs, and more tailored information and resources for the target population [45, 46]. Social media, and in particular Social Networking Sites (e.g., Facebook) are a novel form of technology that can be leveraged for health interventions [47]. Facebook in particular has been utilised in successful interventions targeting a range of health behaviours including smoking cessation [48], stress reduction [49], sexual health promotion [50], and physical activity [51]. Although social media is generally considered to be a risk factor for developing poor body image [52], there may be opportunities to utilise this platform as part of the solution. For example, a study examining the impact of exposure to body positive posts on social media found that this content was associated with improved mood and increased body appreciation and satisfaction among young women [53]. One other study has also used Facebook as an additional element in the delivery of a self-compassion intervention and was successful in improving body appreciation and self-compassion [54]. These findings suggest that disrupting social media, so that it becomes a place where body positive messages are promoted, may be an important step in combating the impact of unrealistic media ideals on body image [55, 56].

Many mothers already interact with social media, and access Facebook to engage with friends, seek support, and offer support to others [57]. As such, Facebook is an appropriate platform to deliver health promotion interventions for mothers. Social networking platforms leverage aspects of online social support and provide the opportunity to deliver intervention material in a flexible manner suited to a mother’s new family commitments [58]. The option for the use of private group functionality on Facebook also allows participants to engage in posting information and personal reflections in a way that preserves a degree of anonymity and may reduce the risk of public shaming [39].

The current research aimed to utilise a design thinking approach to conduct a feasibility and acceptability study, exploring whether evidence-based body image resources delivered online via a five-day Facebook Challenge are well received by mothers, and whether there is qualitative evidence of change in relation to body appreciation and the reduction of thin-ideal internalisation among challenge participants. Feasibility studies are recommended by the British Medical Research Council [59], particularly in the development of complex health interventions.

## Methods

Ethics approval was provided by Flinders University ethics committee (Project: 8334) with mirror approval obtained from Victoria University (Project: 8334) and the University of Queensland (Project: 2020000367). All methods were performed in accordance with the relevant guidelines and regulations as stipulated by the Universities providing approval. Informed consent for study participation was obtained from all participants, as indicated by their completion of the questionnaire and posting in the group.

Participant recruitment was conducted online via Facebook and Instagram and through the Body Confident Mums (BCM) website. Multiple posts advertised the challenge to mothers using organic reach, and $1000 AUD of paid advertising targeted mothers in Australia who were interested in parenting and health content. Mothers interested in joining the challenge were asked to join a private Facebook group and completed screening questions of a) How many children do you have? b) What age(s) is/are your child/children? And c) What is your town/state/country? Mothers were then provided with a PDF Participant Information Sheet about the study and were accepted to join the group.

### Intervention

The Body Confident Mums challenge was delivered over a five-day period starting on a Monday, with participants receiving one instructional video, one body image task, and one reflection prompt, each day. During the challenge, a Facebook live video from the lead researcher (last author) was released outlining the task for the day, how participants might benefit, and the importance of participation by posting on the group’s discussion board. Shortly after the video was uploaded, the instructions for the day’s task were released as a separate post providing relevant links and reflection prompts. See Table 1 for a summary of posts and discussion.

**Table 1:** Content of the Body Confident Mums 5-day Challenge

Day	Task	Reflection Prompts
1	**Thinking self-compassionately** Self-Compassion Recording specific to self-compassion around the postpartum body	• Introduce yourself and the age of your kids and if you like, share a photo of something that represents your mama journey. • Share what part of your body have you been appreciating lately, and why
2	**Thankyou body** Functionality journal writing prompts around appreciation of senses, and what your body can do physically. Write down three things that you are particularly grateful for about your body today.	• How does it feel to talk about the good parts of our bodies, and why might this be? • How did this exercise make you feel, and why might that be?
3	**Embracing body functionality** Functionality journal writing prompts around appreciation of internal body functions and creative endeavours. Write down three things that you are particularly grateful for about your body today.	• What would you say to your younger self if you could write her a letter, knowing what you know now? • What could you say to yourself in moments where you might be feeling bad about your body, and if you don’t feel this way, what could you say to someone else, that incorporates self-compassion, gratitude, and embracing body functionality…
4	**Thinking self-compassionately about parenting** Self-Compassion Recording specific to self-compassion around parenting	• What is an aspect of being a mum that you do really well and why does it matter to you? • What are some mum-guilts or external expectations that you can let go of?
5	**Role Modelling Body Confidence** One-page list of tips for role modelling positive body image to children	• What is something that will you work on in terms of role modelling body confidence for your kids? • What have you learned from engaging in this challenge?

To increase the effectiveness of materials, challenge tasks were derived from existing evidence-based interventions, then adapted to be specific to mothers. Two self-compassion mindfulness audio recordings were created and recorded by the research team, adapted from scripts adapted from Neff and Germer [42] Loving Kindness audio files to relate specifically to motherhood and body image. Two body appreciation journaling tasks were adapted from the Expand Your Horizons program [60] with minor wording changes to emphasise the post-partum body and motherhood experience. To target the role modelling aspect of the challenge, a one-page list of tips for role modelling positive body image to children was adapted from the Confident Body, Confident Child program [61, 62]. This document captured evidence-based strategies for parents to role-model positive behaviours around body image, eating habits, and physical activity [63]. Reflection prompts were also included after each task, and these were designed in alignment with cognitive dissonance frameworks [30].

The first and last authors facilitated social discussion within the Facebook group, encouraged comments amongst group members, and “liking” all the posts of participants. At the end of Challenge on the fifth day, an additional message was posted to the group inviting participants to provide more formal feedback on the content and resources via a Qualtrics survey. One week later, a final reminder was published to encourage people to complete the survey if they had not yet done so.

Prizes were used to encourage participants to engage in the challenge content and provide feedback. These included activewear, a book, and parenting resources which were donated by small businesses as prizes for engagement in challenge content. Prizes were allocated based on the most frequent contributors as determined by Facebook metric data. $100 gift cards were used to thank participants for completing the feedback survey, which were allocated based on a random number generator. Prize winners were announced in the group and sent to participants within 1 month following the end of the challenge.

### Measures

All of the posts provided by participants during the intervention were collected as qualitative data, which were examined for evidence of changes in body appreciation, internalisation of the thin-ideal and overall feasibility and acceptability. In addition, a Qualtrics survey was designed to capture additional feedback about how practical, appropriate, and enjoyable participants found the resources delivered throughout the challenge. At the conclusion of the challenge, details of the Qualtrics Feedback survey were posted within the broader group, and a smaller group of women (*n* = 22) completed this survey. Items were developed specifically to gather early-stage feedback from end-users. Participants were asked to choose on a Likert scale (1 = Strongly Agree to 5 = Strongly Disagree) to what extent they agreed with a series of statements. For example, participants were asked to respond to the following: (1) I enjoyed the challenge; (2) I learned a lot in the challenge; (3) I enjoyed posting to the group in the challenge; (4) I found the challenge easy to navigate and; (5) the pace of the challenge was appropriate. A full list of these items are included in the results in Tables 3 and 4.

### Data preparation and analysis

Following the conclusion of the Challenge, the text from all posts and comments from participants were extracted from the Facebook group into a separate document for analysis. Accompanying photos were not copied and participant names were removed to preserve participant confidentiality.

Qualitative analysis was used to explore and identify themes in data collected from the Facebook Challenge. Qualitative analysis was conducted according to the six-step process outlined by [64]. Facebook posts and comments from group members were copied into a Microsoft Excel worksheet and formatted to ensure each singular idea, comment or feedback item was presented as a separate line item.

Initial coding was recorded by the first author, capturing each singular idea or comment as a short phrase. This formed the initial theme list reflecting saliency of key points in the main data set. Several rounds of iterative re-coding then occurred to facilitate the best representation of data categorised into a set of themes to answer the research questions. A second independent coder reviewed the original list of codes and final set of themes.

Survey data was analysed using SPSS version 26. Descriptive data analyses were used to obtain results. Open-ended comments were analysed using indictive thematic analysis [64] using the process outlined above.

## Results

All *N* = 120 mothers who requested to join the BCM Challenge group were accepted into the group and had children who ranged from 8 weeks old to 12 years old. Twenty-two participants completed the Qualtrics feedback survey. Mothers who completed the feedback survey were an average of 35.3 years old (*Standard Deviation*, *SD* = 5.17). Half (50%) had two children, 36% had one child, and 14% had three children. In reporting the age of their youngest child, half of the participants (50%) indicated 1–2 years, 29% less than 12 months, 20% 3–4 years, and 1% more than 4 years old.

### Thematic analysis of Facebook group content

Engagement in the BCM Challenge was determined based on a range of metrics. *N* = 120 mothers joined the Challenge, and there were *N* = 527 individual engagements across the week (post likes, post comments, individuals’ posts, other interactions). In total, there were *n* = 180 participant responses to challenge prompts, including comments between participants. The number of responses to challenge prompts posted by individual participants declined across the week, from day 1 (*n* = 24), day 2 (*n* = 17), day 3 (*n* = 10), day 4 (*n* = 10), today 5 (*n* = 9). Responses were analysed for themes that emerged, and these are presented below and summarised in Table 2.

**Table 2:** Thematic analysis summary – themes of challenge participant outcomes

Theme	Sub-themes	Example quote
1 Internal shift in mindset	1a. Body appreciation	“I really appreciate my breasts as they have feed & comforted my youngest daughter for two and a half years & still going strong, which I am so proud of”
	1b. Focus on body function	“I like my legs - they get me around everywhere, I love walking and running at the beach, they are getting stronger with running”
	1c. Gratitude	“I am grateful for my eyes, I don’t need glasses(yet) and they let me see the beautiful lives I created. No matter how tired I am they still open in the middle of the night when I’m needed. I love watching my babies grow”
	1d. Positive re-framing	“It’s ok to feel overwhelmed as a parent (it’s relentless) and being a good mum is not synonymous with being perfect”
	1e. Shift in mindset	“I feel a shift in my mindset is happening - that I am beginning to appreciate my body more”
2 External shifts in perspective	2a. Reclaiming body	“Talking about what my body can do and appreciating it felt like reclaiming my space, reclaiming my body and honouring it in a way. It helped me drop the resentment and feel like this is my body, it’s my choice and I choose to be here”
	2b. Role-modelling	“Learning to love my body and what it can do is the best way I can help my children love themselves”
	2c. Focus on the bigger picture	“The creases around your eyes reflect the laughter and smiles you’ve had over the last 3 decades”
3 Change in how I treat myself	3a. Pride in self	“Instead of seeing what I dislike, I see what my body has achieved and makes me feel proud. It shows me that my body is more than just the negative parts that I see of it”
	3b. Self-care	“I’m finally starting to learn that in order for my girls to have the happy and healthy mum they deserve, I need to take care & look after myself too”
	3c. Self-compassion	“You’ve done your best. You are loved. You are making a difference. This is your journey and no one expects perfection”
4 Increased self-awareness	4a. Internal bodily awareness	“At the end of the day when I could choose to do this task or go to bed early, I listened to my body”
	4b. Mindfulness	“If I feel bad about part of my body I acknowledge this feeling without judgement and move on”
	4c. Self-awareness	“I realised I hadn’t given much thought to my body other than how it looks, or when it doesn’t work properly. What about all the amazing things it does do?”
	4d. Self-reflection	“It’s so funny to actually put thoughts onto paper - it’s makes you think and question what you’re actually saying”

#### Internal shift in mindset

Throughout the duration of the challenge, participants appeared to experience a shift in their thinking about their bodies. The women were surprised and delighted that it was possible to see themselves through a different and more constructive lens. For example, one mum posted in the group, “I’m used to having a narrow focus on the attractiveness of my body, without much thought about what my body can do. It was great to see the value of my body beyond what it looks like”. A particular focus in this theme was participants’ appreciation for their body and through this, a newfound view of their body as valued and valuable: “I never thought I’d say this, but I have been appreciating my soft squishy curves lately. Good for giving comforting cuddles to my boys!”. A particular focus of the shift was the recognition that participants’ bodies, their features and imperfections, told a story about their life. Their body was their home and had supported them through all of their experiences and memories including the monumental event of giving birth and nourishing their child(ren). This reflective process brought about positive change and a sense of relief among the mothers, because now they did not have to conform to societal expectations, and could think of their body in a different light. A participant posted to the group something she wanted to remind herself, “Remember you housed two babies in that belly and what happened on the inside is far more significant than what you see on the outside”. These statements often led participants to reflect on how grateful they were for their bodies, particularly in relation to how their body connects them to their children, and also a broader recognition that their body was worthy and deserving of attention, compassion, and care.

#### External shift in perspective

During the challenge, participants appeared to, evaluate how their body image concerns fit within the context of their broader life priorities. Thinking about one’s body in the context of what was truly important to, or valued by, the participants allowed a new line of thinking to emerge. Participants reflected on the ‘bigger picture’ and what they wanted their life to be about. For many, this sparked a conversation that reflected all the things that made life fulfilling and meaningful. One mum shared what parts of life are most important, “listening to my two-year old’s funny stories, feeling my unborn baby move, tasting delicious food, being in my garden watching the progress of the apricots on the tree and smelling the spring flowers”. These reflections seemed to reinforce the things in life that brought participants joy and meaning, all of which had nothing to do with their appearance. Another participant suggested to the group, “don’t waste time dieting and weighing yourself everyday!” and, “enjoy your food, enjoy yourself, it goes too… fast”. When they gave themselves time and space to reflect on their body concerns and what was personally important, they discovered that appearance was not high on their priority list. Instead, it was the importance of loving themselves and being present for their families. This seemed to suggest spending time negatively obsessing over body parts diverted time and energy away from what was most valued. A group member publicly reflected, “for me this is a great reminder to not let feelings about my body get in the way of being present for my kids”.

As participants continued to think about the role of body image in their life, a strong motivation for change emerged. Almost all participants commented on the importance of wanting to be a good role-model for their children. They did not want their current behaviours (e.g. deciding not to go swimming because of body concerns) to impact their children now or in the future (e.g. growing up to adopt negative body behaviours). One participant proclaimed to the group, “Wear the bloody cossie. Be brave” (Note; Cossie is an Australian term referring to swimwear). Another mentioned it was important for her kids “to have happy memories and see their mum’s fun and happy side”. Participants seemed to weigh up the costs and benefits of focusing negatively on their own body versus fully engaging with their lives. One mum commented that, “learning to love my body and what it can do is the best way I can help my children love themselves”. Overall, body image concerns were de-prioritised, and became less important among participants than loving their bodies and being a positive role-model for their families.

#### A change in how I treat myself

Another prominent theme reflected a powerful sentiment in how participants spoke to themselves and treated themselves more broadly. First there was a sense that participants were able to identify that they could engage in more self-compassion in both their internal dialogue but also in terms of self-care and looking after their mental and physical health and well-being. This was a challenging task for many participants, as many realised it had been some time since they had last been nice to themselves or looked after themselves. A transition was evident however where participants began to realise that to be the best version of themselves for their children, they had to invest in themselves too. One mum shared*,* “I’m finally starting to learn that in order for my girls to have the happy and healthy mum they deserve, I need to take care of and look after myself too. I know I need to let go of that guilt because as mums it’s important to look after ourselves too”. These reflections were often framed as statements of intent to change, indicating participants were really considering change and the ways in which they could better practice self-compassion and self-care. This trend was also identified in a broader sense when participants reflected on their own parenting abilities. Participants openly shared what they did well, and even offered ways they might be able to drop any guilt around their parenting practices. This shift was recognised through parents reflecting that they were often able to perceive their own parenting failings but rarely recognised their successes. This challenge provided an opportunity for participants to reflect on what parts of parenting they did well, and to develop compassion toward imperfect parenting. One mum posted to the group, “could let go of so much mummy guilt! At the moment I’m particularly struggling with the feeling that I’m not giving my boys enough individual attention. If only there were two of me to meet all of their needs... but I’m doing the best I can”.

Lastly, these changes in treating oneself with kindness, respect and compassion filtered into intentions to build positive body behaviours including intuitive eating and joyful movement. A participant shared her personal reflection: “I’ve learnt that when I am kind to my body, I look after it (I eat healthier and love the long walks it can do)”. Similarly, another participant shared, “I will work on joining in with my son’s joyful physical movement, he loves moving - especially running and jumping - and he would love it even more if I joined him”*.* Of note, is that these positive behaviour change intentions were driven from a place of self-compassion and self-care rather than an emphasis of losing weight or changing appearance.

#### Increased self-awareness

Participants engaged in significant self-reflection, a process which led them to develop greater awareness about their thoughts, feelings, behaviours, and needs. Group members seemed to explore their appraisals about themselves and their parenting on a completely different level than they would otherwise. One mum reflected, “having a set time to write…pushed me past the initial layer of thoughts and gave time for deeper layers of awareness to emerge…after the first couple of minutes, that’s when I had more realisations about myself”. This process seemed to trigger an increase in self-awareness and supported a more mindful approach to meeting emotional and physical needs such as self-care (e.g., taking time to go to yoga). Mums tended to agree that they often did not have time to self-reflect, and it had been a long time since they had given themselves this opportunity. Participants remembered how good it felt to give themselves this reflection space, many committing to engage in self-reflective acts more often. Through this process of increasing levels of self-awareness, participants learnt new things about themselves and new strategies to promote positive body image that worked for them. A group member posted: “If I feel bad about a part of my body, I acknowledge this feeling without judgement and move on. I also think about what am I going to do for myself today?”. It was evident that participants had the opportunity to really consider how they had been treating themselves and realistically assess how they viewed themselves and what they might want to change.

### Results from the feedback survey

#### Acceptability and perceived enjoyment of the challenge

The Qualtrics feedback survey was completed by a smaller subset of women who completed the challenge (*n* = 22). Participants were asked general questions about their enjoyment of the challenge content and perception of the effect of the content on their mood and body image. Findings are summarise din Table 3. The majority of participants agreed that they enjoyed the challenge, were willing to recommend it to others, and were interested in continuing the activities after the challenge was complete. Whilst the majority enjoyed posting in the group environment, a small proportion (6%) indicated that they did not like posting to the group.

**Table 3:** Participant (*n* = 22) survey feedback on enjoyment and effects of intervention activities

	Strongly agree or somewhat agree	Neither agree nor disagree	Somewhat disagree or strongly disagree
I would recommend these activities to others	100%	0%	0%
I enjoyed the challenge	94%	6%	0%
I would like to do more of these activities	94%	6%	0%
I enjoyed the activities	94%	6%	0%
I learned a lot in the challenge	88%	12%	0%
The activities made me feel better	88%	12%	0%
I enjoyed posting to the group in the challenge	76%	18%	6%
The activities made me feel worse	0%	12%	88%
Information about role modelling positive body image had an impact for me	94%	6%	0%
Self-compassion recording about being kind to yourself as a mum had an impact for me	89%	12%	0%
Self-compassion recordings about being kind to your body had an impact for me	88%	12%	0%
Journaling about the parts of your body that you are grateful for had an impact for me	82%	12%	6%

In the Survey, participants were also asked specific questions about the practicality of the challenge delivery. These findings are reported in Table 4.

**Table 4:** Participant (*n* = 22) feedback on intervention delivery

	Strongly agree or somewhat agree	Neither agree nor disagree	Somewhat disagree or strongly disagree
There was enough information to complete the activities	94%	0%	6%
I found the challenge content easy to navigate	89%	6%	5%
The pace of the challenge was appropriate	71%	12%	18%
The activities required an appropriate amount of time	70%	12%	18%
Reading other people’s posts in the group had an impact on me	89%	12%	0%
Facebook Live updates had an impact on me	88%	12%	0%
Posting publicly in the group had an impact on me	87%	0%	13%

The Qualtrics survey also allowed participants to provide additional open-ended feedback. A common theme in these responses was that participants reported the use of Facebook as helpful. One participant shared, “Using Facebook made it easy to access on my phone and I was familiar with the platform. I loved the mix of private activities and sharing on the discussion board in the units. I also appreciated that each unit had written instructions as well as discussion in the video”. One mum commented that, “reading other people’s posts (was) one of the best parts of the challenge for me…it’s the writing it down, and reading others that helped it really sink in”. Another mum shared that “there is value in posting to the group as it encourages vulnerability but in a safe environment”, and another mum wrote, “I love that we got to read about other Mums and the challenges they face. Made me feel less alone”.

Some participants mentioned that they struggled with the timing of activities and pace of the overall challenge. One mum shared, “I…struggled to find the time to do this every day. Perhaps giving a couple of days for each challenge would be easier for working mums”. In contrast other mums commented that, “The activities were very achievable and highlighted to me the importance of taking those 10 mins out”.

## Discussion

This study aimed to assess the feasibility, acceptability, and response to a five-day evidence-based body image intervention delivered to mothers, via Facebook. Qualitative analysis of the posts by women during the challenge led to identification of four themes, (1) Internal shift in mindset; (2) External shift in perspective; (3) A change in how I treat myself; and (4) Increased self-awareness. These themes indicated a likely shift in body image through known processes of change relating to self-compassion and appreciation of body functionality, which may in turn effect internalisation of the thin-ideal, and improve body dissatisfaction according to the Tripartite model for postpartum women [65].

Results of a survey with a smaller portion of participants indicated that they enjoyed the challenge, found it to be useful, and would recommend it to others. However, there were issues with the time commitment, and the pace of the challenge, with some women suggesting the challenge be spread out a little more.

### Feasibility and acceptability

Rich data collected from this study demonstrated that delivery of body image resources focusing on self-compassion, body functionality appreciation, gratitude, and mindfulness were mostly feasible and acceptable with a population of mothers utilising a Facebook private group as a delivery platform. Feasibility and acceptability was further supported by a small sub-set of survey participants that agreed using Facebook as a tool to deliver the challenge was easy, flexible, and functional. This supports previous research that has used private group functionality on Facebook as an effective and viable platform to deliver health resources and interventions [55, 66].

Participants agreed the content was enjoyable, engaging, and had a positive impact. Mothers indicated that the challenge activities had a positive impact on their wellbeing, and reported that the act of posting reflections, engaging with others in the online community and watching the daily videos was helpful. Similar to an online parenting intervention by Swindle, Ward, and Whiteside-Mansell [67], the combination of receiving appropriate resources, and then being able to practice and reflect within a supportive group environment may be important for participant acceptability and responses to the intervention.

### Responses to the intervention

There was qualitative evidence that participation in the Challenge may have improved body appreciation, based on participants’ comments and their intention to change attitudes and behaviours in relation to body functionality, improved self-compassion, and reduction of internalisation of the thin-ideal.

A major finding was that participants appeared to have engaged in an internal shift relating to how they viewed themselves. This occurred through a focus on body function over appearance and acknowledging bodily changes as a result of pregnancy, giving birth or breast-feeding that brings increased function and meaning to their body, rather than focusing on aesthetic changes. These findings are consistent with that of another journaling intervention [68], where the development of body focused gratitude also appeared to play an important role in reframing negative views of self, leading to an overall improvement in body appreciation. Similarly, quantitative evaluation of the *Expand Your Horizons* program [60] that focuses on body functionality also report enhanced body appreciation [35, 69, 70]. It may be of particular importance to practice gratitude toward body functionality, as opposed to body appearance, to bolster positive and sustainable improvements in body appreciation. It is possible this has added benefit for women in the postpartum period who are experiencing significant changes to the appearance and function of their body [71].

Posts and comments in the group also indicated that participants were starting to treat themselves with more self-compassion in relation to their bodies and their parenting. Participants seemed to experience an increased awareness of negative internal dialogue and a demonstrated willingness to respond to oneself with kindness and appreciation of functionality. This outcome supports the previously established link between mindful self-awareness, self-compassion and overall body image outcomes, and may indicate self-awareness is an important precursor to developing self-compassion [72–74]. The literature also suggests self-compassion is a strong mediator of reducing internalisation of the thin-ideal [39, 75], and our research further supports this through being able to accept oneself despite not meeting societal thin norms. It is possible that self-compassion was the mechanism by which individuals were able to let go of body image concerns and negative appraisals of self-image and to re-focus attention on other, more meaningful aspects of life in this intervention.

A unique aspect of the challenge, afforded by the use of a social networking platform to deliver intervention content, was that participants posted reflections to a private Facebook group, and were able to read and comment on the reflections of others in the group. Posting these reflections of intentions to change and body appreciation may have facilitated a state of cognitive dissonance as participants publicly and voluntarily entered into statements of counter-attitudinal advocacy toward body appreciation and self-compassion [31, 76]. The process of cognitive dissonance is in line with evidence within the literature for supporting health behaviour change related to body dissatisfaction and eating disorder prevention [30, 31] and broader public health targets [55, 77, 78]. Additionally, collective rejection of the thin-ideal through social norms, peer support, and validation was likely to be a facilitator of change, highlighting the importance of social support when making attitudinal and behaviour change in the context of body image [79]. The Facebook group environment, where positive social validation is present, may be important especially in short-term interventions to build self-compassion and reduction of the thin-ideal.

### Strengths and weaknesses

This is the first study to describe a combination of self-compassion and body functionality approaches to improving body image among mothers, and the delivery of a body image intervention using Facebook. Strengths of the study include investigation of a body image intervention with a unique population and pilot testing of the delivery of evidence-based programs delivered using a social networking platform. The qualitative data collected during the intervention provides an in-depth and rich understanding of how people engaged with the challenge in the online closed group setting. Two researchers were involved in thematic analysis and the coding process to reduce potential bias and to ensure themes were reflective of raw qualitative data.

This study had a number of limitations, which are important to acknowledge. One major limitation was the low participation rate in the daily challenge tasks among the 120 participants enrolled, and attrition across the 5 days of the Challenge. Given that this intervention was delivered via Facebook, the delivery of the content to participants’ feeds was not in the control of the researchers and was subject to the algorithms determining feed content. As such, some participants may not have seen challenge content, or calls for participation in the feedback survey. It is possible that many of the participants who joined the challenge did view the instructions and engage with the tasks offline, but did not post to the group, perhaps due to feelings of vulnerability or wanting to maintain privacy. Further, only 22 participants completed the feedback survey, and these were likely to be the participants who remained engaged at the end of the challenge when the invitation to complete the feedback was posted. These participants may therefore represent a biased sample of mothers who engaged with and enjoyed the challenge, while others simply did not engage, did not respond positively or discontinued. Future research could overcome these limitations by using quantitative measures of body image prior to initiation of the Challenge, and again at post-intervention and follow-up, whilst also allowing participants to post in the group format or to complete tasks offline (but provide data on whether or not this was done). This would facilitate an understanding of whether engagement leads to body image changes on validated measures and is moderated by levels of engagement in the intervention materials. Another important limitation is that very little information on participant demographics was collected, and we are therefore unable to say how generalisable these results might be to a broad population, and especially to mothers outside of Australia. Lack of quantitative data also meant additional factors which are known to correlate with body image disturbance, including BMI, maternal age and weight prior to falling pregnant were unable to be assessed.

Despite these important limitations, the present study still provides important insights into the feasibility of online Facebook interventions for mothers. It is critical that researchers utilise rich sources of feedback for early pilot testing of interventions to gather feedback from end-users that can facilitate iterative product development to create interventions that people are more likely to engage with, and enjoy [80].

## Conclusions

The outcomes from the Body Confident Mums challenge indicate that the body image intervention delivered online was appreciated by mothers, and may have led to changes in their attitudes and behaviours. Closed Facebook groups may be a viable platform for the delivery of health promotion programs. Analysis of the responses to reflection prompts, and feedback provided by participants indicated that the online environment afforded by the private group function on social media may be particularly useful in creating attitudinal and behavioural change through counter-attitudinal advocacy and creating a shift in social norms by reading the experiences of others. Qualitative evidence from participants’ engagement in the intervention indicates improvements in body appreciation with additional confidence gained in body confident parenting practices. Iterative development of this intervention, informed further by feedback from end-users is recommended, and once the product is in its final form, a full scale RCT is recommended to assess the efficacy of this intervention.

## Data Availability

It is not possible to share the qualitative or quantitative data used in this project publicly, as this was not what participants agreed to in the participant information and consent form. Data can be made available from the corresponding author on reasonable request.
